# First Genome Sequence of a Syntrophic Acetate-Oxidizing Bacterium, *Tepidanaerobacter acetatoxydans* Strain Re1

**DOI:** 10.1128/genomeA.00213-12

**Published:** 2013-02-21

**Authors:** Shahid Manzoor, Erik Bongcam-Rudloff, Anna Schnürer, Bettina Müller

**Affiliations:** a Department of Animal Breeding and Genetics Science, Swedish University of Agricultural Science, Global Bioinformatics Centre, Uppsala, Sweden; c Department of Microbiology, Swedish University of Agricultural Sciences, Uppsala BioCenter, Uppsala, Sweden; b University of the Punjab, Lahore, Pakistan

## Abstract

Syntrophic acetate-oxidizing bacteria (SAOB) have been identified as key organisms for efficient biogas production from protein-rich materials. *Tepidanaerobacter acetatoxydans* is the first reported SAOB for which the genome has been sequenced. Genome analysis will aid us in understanding the mechanisms regulating syntrophy, particularly energy-conserving and electron transfer mechanisms.

## GENOME ANNOUNCEMENT

*Tepidanaerobacter acetatoxydans* strain Re1 is the type strain of a total of four anaerobic syntrophic acetate-oxidizing bacteria (SAOB) isolated from ammonium-rich, mesophilic, methanogenic systems (1). It is Gram positive and belongs within the *Firmicutes* phylum to the class of *Clostridia*. It shares 96% 16S rRNA gene sequence identity with the known *Tepidanaerobacter* member *T. syntrophicus* (2). It is rod shaped, motile, and spore forming and has been found to be both thermotolerant up to a temperature of 55°C and ammonium tolerant up to a level of 1 M NH_4_Cl (1). Growth experiments and genetic studies allocated *T. acetatoxydans* to the physiological group of homoacetogens producing acetate through the Wood-Ljungdahl pathway when growing heterotrophically (1, 3). However, when growing in syntrophy with methanogens, SAOB supposedly reverse this pathway and oxidize acetate to hydrogen and carbon dioxide (3–5). Comparative genome analysis including nonsyntrophic homoacetogens and syntrophic nonhomoacetogens might reveal general features mediating syntrophic competence. It will throw light on the biochemical and regulatory mechanism behind the syntrophic acetate oxidation pathway and will enable further investigations of the activity of SAOB in different environments. The genome of the *Tepidanaerobacter acetatoxydans* strain Re1 was sequenced by using Ion Torrent PGM Systems. Low-quality and short reads were filtered, and the remaining 3,333,516 reads were assembled with Newbler 2.8 by the *de novo* assembly method. A total of 3,126,071 reads (95.72%) were aligned to produce 96 scaffolds. The average length of contigs was 25.5 kb, resulting in a total length of 2,759,867 bp. The final approximate coverage for these contigs was about 209×. The genome sequence was annotated with different annotation tools. Open reading frames (ORF) were predicted by MaGe (Microbial Genome Annotation and Analysis Platform). rRNAs were predicted by using RNAmmer (6), and tRNAs were identified with tRNAscan-SE 1.21 (7). A total of 2,701 ORF were predicted, including 2,524 protein-coding sequences; 52 tRNAs; 2 copies each of 5S RNA, 16S rRNA, and 23S rRNA; 3 copies of ncRNA; and 1 copy of tmRNA. The total genome has a G+C content of 37.5% along with an average coding sequence (CDS) length of 917 bp, an average intergenic length of 136.12 bp, and a protein-coding density of 86.92%. Further detailed analysis, including functional annotation, comparative genomics, and metabolic pathways analysis at the genome scale, is in process and the results will be included in our future publication.

### Nucleotide sequence accession number. 

The genome sequence of *Tepidanaerobacter acetatoxydans* strain Re1 has been deposited in the GenBank database with the accession number HF563609.
